# Genome-scale model reconstruction of the methylotrophic yeast *Ogataea polymorpha*

**DOI:** 10.1186/s12896-021-00675-w

**Published:** 2021-03-15

**Authors:** Ulf W Liebal, Brigida A Fabry, Aarthi Ravikrishnan, Constantin VL Schedel, Simone Schmitz, Lars M Blank, Birgitta E Ebert

**Affiliations:** 1 Institute of Applied Microbiology-iAMB, Aachen Biology and Biotechnology-ABBt, RWTH Aachen University, Worringer Weg 1, Aachen, 52074 Germany; 2 Genome Institute of Singapore, 60 Biopolis Street, Genome, 03-01, Singapore, 138672 Singapore; 3 Australian Institute for Bioengineering and Nanotechnology, The University of Queensland, Brisbane, QLD 4072 Australia; 4 CSIRO Future Science Platform in Synthetic Biology, Commonwealth Scientific and Industrial Research Organisation (CSIRO), Black Mountain, ACT 2601 Australia

**Keywords:** Biotechnology, Genome-scale metabolic model, Metabolic reconstruction, Metabolic engineering, COBRA, Methylotrophy

## Abstract

**Background:**

*Ogataea polymorpha* is a thermotolerant, methylotrophic yeast with significant industrial applications. While previously mainly used for protein synthesis, it also holds promise for producing platform chemicals. *O. polymorpha* has the distinct advantage of using methanol as a substrate, which could be potentially derived from carbon capture and utilization streams. Full development of the organism into a production strain and estimation of the metabolic capabilities require additional strain design, guided by metabolic modeling with a genome-scale metabolic model. However, to date, no genome-scale metabolic model is available for *O. polymorpha*.

**Results:**

To overcome this limitation, we used a published reconstruction of the closely related yeast *Komagataella phaffii* as a reference and corrected reactions based on KEGG and MGOB annotation. Additionally, we conducted phenotype microarray experiments to test the suitability of 190 substrates as carbon sources. Over three-quarter of the substrate use was correctly reproduced by the model and 27 new substrates were added, that were not present in the *K. phaffii* reference model.

**Conclusion:**

The developed genome-scale metabolic model of *O. polymorpha* will support the engineering of synthetic metabolic capabilities and enable the optimization of production processes, thereby supporting a sustainable future methanol economy.

**Supplementary Information:**

The online version contains supplementary material available at (10.1186/s12896-021-00675-w).

## Background

*Ogataea polymorpha* (*Hansenula polymorpha*; *Pichia angusta*) is a widely used yeast for biotechnological applications. It is environmentally ubiquitous and has been isolated, among others, from orange juice, maize meal, and insect guts [1]. The ability to metabolize methanol stems from its close association to the phyllosphere [2]. *O. polymorpha* has some unique features among methylotrophic yeasts such as high growth rate, temperature tolerance, and nitrate fixation [3] and has been optimized to express peptides and proteins [4]. A particular benefit for biotechnological applications is the lack of byproducts even on high glucose feeds [4, 5]. Furthermore, the conversion of methanol to succinic acid via methylotrophic yeasts was found to provide a competitive alternative to conventional petrochemical approaches in a computational comparison of various microbial carbon fixation strategies [6].

Methylotrophy in yeast is restricted to a single multigenus clade, with its most prominent members being *Komagataella phaffii*, *O. polymorpha*, and *Candida arabinofermentans* [7]. The phylogeny of methylotrophic yeast is complex and subject to recent updates [8]. The *Ogataea* strains most frequently mentioned in the literature are DL-1 (ATCC 26012), CBS4732 (ATCC 34438, NRRL-Y-5445, TB-3) and NCYC 495 (ATCC 14754, NRRL Y-1798) [1, 9, 10]. The genomes of all three strains have been sequenced and annotated [1, 7, 10]. A genomic comparison of *O. polymorpha* DL-1 and *K. phaffii* showed that they share three-quarters of enzymes whereas the non-overlapping proteome contains mostly hypothetical, uncharacterized proteins, indicating their close relationship and overlapping metabolic features [10].

The methylotrophic model organism *K. phaffii* is one of the few yeasts that have the ability to use methanol as the only carbon source for energy production. *K. phaffii* was developed into an effective producer of recombinant proteins based on the strength of the native methanol-responsive promoter expression system. The wealth of knowledge has lead to the development of metabolic models on the genome level (GSMM) for simulation and strain engineering [11, 12]. The most recent models are iMT1026v3 and iRY1243 [13, 14]. Based on the close relationship among methylotrophic yeasts and the availability of sequencing data in genomic databases, the development of a GSMM for the metabolism of *O. polymorpha* is desirable.

*O. polymorpha* and *K. phaffii* share many similarities especially regarding their methanol metabolism. However, several metabolic features are distinctive. *O. polymorpha* is capable of growth at 50^∘^C and is therefore one of the most thermotolerant eukaryotic microorganisms [15]. In contrast to *K. phaffii*, *O. polymorpha* can ferment xylose to ethanol [16] and can assimilate nitrate [17]. Moreover, high activity of the AOX promoter with glycerol as the sole carbon source or under glucose starvation is a unique feature of *O. polymorpha*. In *K. phaffii*, activity of AOX strictly depends on the presence of methanol [18].

Here, we present iUL909 as the first GSMM of the biotechnological relevant methylotrophic yeast *O. polymorpha* NCYC 495. The model is based on existing models for *K. phaffii*, extended by species-specific substrate utilization identified in phenotype microarrays. Model predictions of growth rates were found to represent experimental growth in different conditions. We tested the performance for overproduction of lactate, and succinate with methanol and glucose as substrates for biotechnological applications and identified potential targets for amplification of reaction activities.

## Results and discussion

Here, we report on the construction of a genome-scale metabolic model of the methylotrophic yeast *O. polymorpha* named iUL909. iUL909 was generated on the basis of an existing metabolic model of *K. phaffii* iMT1026v3 [13, 14]. Gene identifiers from *K. phaffii* in iMT1026v3 were replaced by those of homologs from *O. polymorpha*. The model was validated against physiological data from substrate utilization tests with phenotype microarrays and shake flask experiments, as well as growth rates from the literature. We further simulated the production of industrially relevant molecules to identify future biotechnological applications. The validity of the SBML model was tested with Memote [19].

### General properties of iUL909

**Fig. 1:**
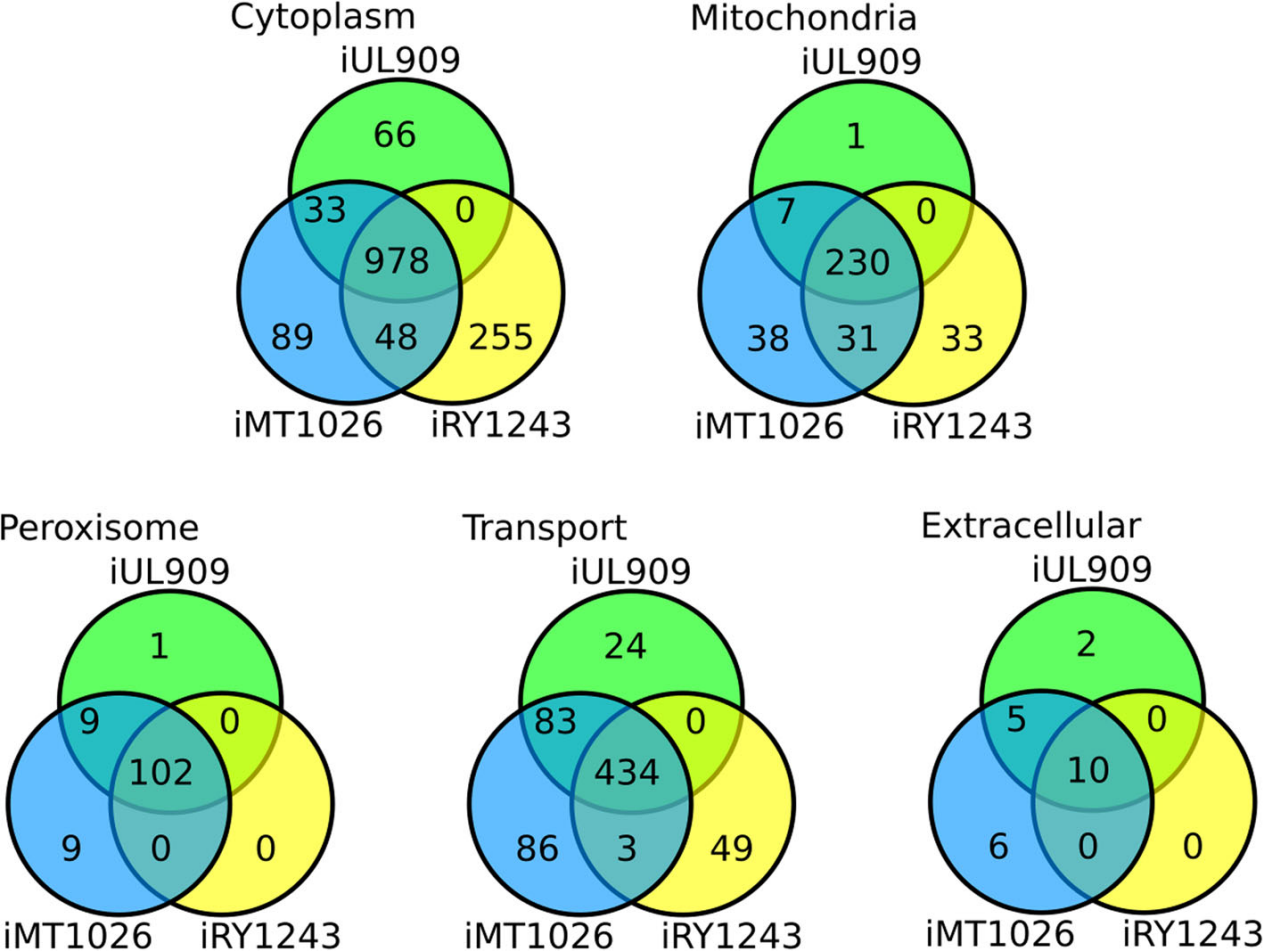
Venn diagrams of compartment specific reactions overlap. The genome-scale metabolic model (GSMM) of *K. phaffii* iMT1026 [13] was used as a reference to construct the GSMM for *O. polymorpha* because of metabolic and phylogenetic similarities (see text). iMT1026 was further adapted for biotechnology in the GSMM iRY1243 [14]. The reactions are fully overlapping for the compartments Golgi, nucleus, endoplasmatic reticulum and vacuole

The close relationship among methylotrophic yeasts is reflected by a high overlap of homologs between *O. polymorpha* and *K. phaffii* [7]. The details of our reconstruction and a comparison with the reference GSMMs are shown in Fig. 1 and Table 1. We mapped genes from *K. phaffii* to *O. polymorpha* using a homolog-search (see Material and Methods section) which failed to find 84 genes (see Additional file 3). However, we added 114 new reactions and 39 new genes (see Additional file 3) from two sources: (i) additionally annotated GPRs from the iRY1243 GSMM, and (ii) genes identified for reactions required for the metabolization of carbon substrates identified with the Biolog® Phenotype microarray. iRY1243 differs from the reference model iMT1026v3 only in reactions associated with transcription/translation, signaling and protein turnover. Since iUL909 covers only metabolic reactions it was not amended with these additional reactions from iRY1243. Overall, the common origin and high overlap of the three GSMMs is visible from the comparable number of the compartmentalized reactions (Fig. 1).

**Table 1:** Feature comparison of the *O. polymorpha* GSMM with respect to the reference GSMM of *K. phaffii*

	iUL909	iMT1026v3	iRY1243
Genes	909	1026	1243
Metabolites	1639	1706	1740
Reactions	2263	2237	2407

### Comparison of iUL909 with iMT1026

**Fig. 2:**
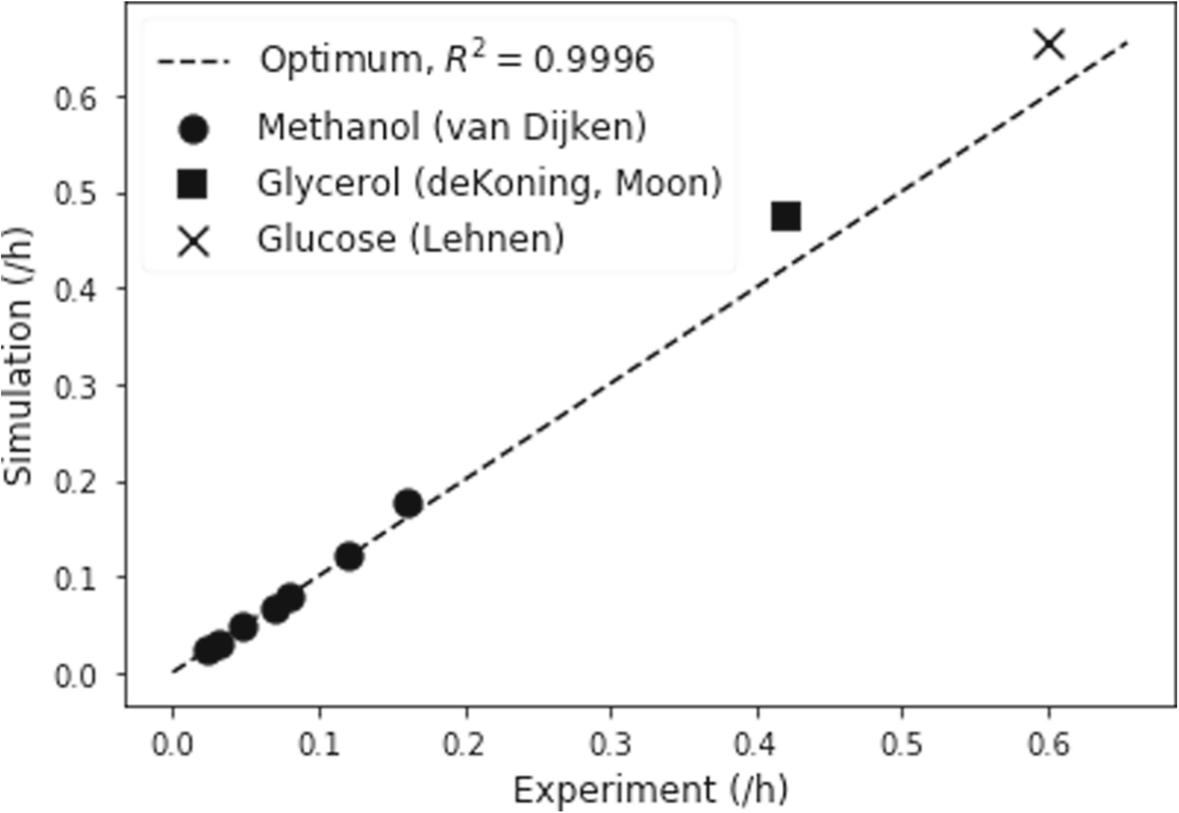
Comparison of experimental and simulated growth rates. Comparison of experimentally measured and simulated growth rates on methanol, glucose, and glycerol. Experimental data were taken from literature [5, 20–22]. Biomass composition, growth-, and non-growth associated maintenance (GAM, NGAM) are based on [13] for the respective substrate

The *O. polymorpha* GSMM reproduces experimental growth rates with growth parameters from iMT1026. All growth parameters were taken from the reference model iMT1026v3, namely growth- and non-growth-associated maintenance energy (GAM, NGAM) as well as the biomass reaction [13]. The substrates tested were methanol, glucose, and glycerol, industrially relevant sources for which literature values of growth rate and substrate uptake rate are available [5, 20–22]. The Pearson correlation coefficient of 0.9996 between predicted and experimental growth rate supports using GAM, NGAM and biomass equation from iMT1026v3 (Fig. 2). In particular, the growth characteristics on methanol reported by Van Dijken et al. [20] for *O. polymorpha* (Fig. 2, circles) is reproduced, underscoring the similarity of biomass composition and methanol metabolism between *O. polymorpha* and *K. phaffii*.

Notwithstanding the high degree of similarity between *K. phaffii* and *O. polymorpha* there were 84 genes in iMT1026 without a detected homolog in *O. polymorpha*. Because many reactions were catalyzed by alternative enzymes, the gene-protein-reaction relationships (GPR) was still satisfied for 62 genes, but 22 gene products were sole catalysts for the associated reactions (see Additional file 3). These 22 reactions lacked a GPR, caused by gene function replacement or loss. Replacement is likely when upstream and downstream reactions are annotated, loss (or misannotation in iMT1026) is likely for orphan reactions without connections to the metabolism. Among those orphan reactions, nine were deleted because they could not carry flux. For example, a potential misannotation was identified for 4 *α*-hydroxytetrahydrobiopterin dehydratase (EC number 4.2.1.96) in iMT1026 for which neither KEGG nor the model itself would allow metabolite flux. However, thirteen reactions were retained because they were metabolically well connected, and their deletion would result in reaction gaps. For example, the identities and the location of all non-proton pumping mitochondrial NADH dehydrogenase system is currently not sufficiently well known. We decided to retain dehydrogenase reactions from the reference model iMT1026. More experiments and data will guide improvements regarding the actual electron and proton homeostasis.

### Substrate identification with biolog plates

Substrate tests with phenotype-microarray plates were conducted to compare predictions of the reference GSMM of *K. phaffii* iMT1026 with the actual growth phenotype of *O. polymorpha*. Biolog’s Phenotype Microarray™ plates test was used to analyze the metabolic utilization of 190 carbon substrates (see Material and Methods section). Table 2 shows the overlap and differences between experiment and simulation run with the *K. phaffii* GSMM iMT1026. Correct growth phenotypes were predicted in 77 *%* cases (13 positives and 134 negatives). In eight cases no growth was experimentally measured, whereas it was predicted by the simulation. Substrates of this class comprised mainly organic acids associated with the TCA cycle (see Additional file 2). We tested growth separately in shake flask experiments and observed growth after two days for all of the eight substrates (Additional file 2). We hypothesize, that the base medium of the Biolog Phenotype Microarray™ was inappropriate for supporting growth with the eight substrates and/or that the cultivation time was too short for achieving adaptation of *O. polymorpha* to the specific environmental condition. Indeed, we verified growth support in shake flask experiments (Additional file 2). There are possibly additional false-negative results among the 134 substrates identified by the Biolog Phenotype Microarrays as non-growth supporting, if, simultaneously, the reference model iMT1026v3 would falsely lack the corresponding metabolic activities. Comparing our growth phenotypes with literature reports [9], we confirmed 17 out of 19 common substrates. As additional carbon sources, we identified raffinose and maltose (see Additional file 2).

**Table 2:** Confusion matrix overview of the growth overlap between *O. polymorpha* in Biolog’s Phenotype Microarray™ plates test and prediction according to the iMT1026v3 GSMM for *K. phaffii*

	iMT1026 growth	iMT1026 no growth
Experiment growth	13	35
Experiment no growth	8	134

Substrates with positive growth not predicted by simulations were added to the genome-scale reconstruction of *O. polymorpha*. As Table 2 shows, *O. polymorpha* grew on 35 substrates although simulations with iMT1026 predicted no growth. Of these previously unsuspected metabolized substrates, we added 27 substrates to the reconstruction iUL909. The remaining eight substrates were omitted because the annotation of metabolic pathways was unlikely. For example alanine amide has no associated pathway, whereas the degradation pathways for fucose and dulcitol involve galactose, which did not display growth in our test and was shown to be not metabolized by *O. polymorpha* [8]. We omitted gentiobiose because we only observed weak growth, although as a D-glucose disaccharide vigorous growth would be expected. *O. polymorpha* grew on L-leucine, and the model had all pathways required for metabolic activity, but a successful growth simulation was technically not achieved.

### Gene annotation and pathway correction

New metabolic features were added and existing reactions corrected. All genes in the reconstruction iUL909 were checked in the methylotroph gene order browser (MGOB) [23] and KEGG [24]. We accessed the UniProt TrEMBL [25] sequence source of *O. polymorpha*^1^ which listed 5167 proteins with only 422 EC number associated enzymes (Additional file 1). Due to the incomplete enzyme coverage by the UniProt annotation we relied on KEGG and the MGOB, and could functionally annotate >97*%* of the genes. We identified enzymes within the biotin pathway converting 8-amino-7oxononanoate to 7,8-diaminononoate while using *S*-adenosylmethionine as a co-substrate. To reconstitute the co-substrate we hypothesized that activity of *S*-adenosyl-4-methylsulfanyl-2-oxobutanoate transaminase (EC number 2.6.1.12) would be present. We corrected the equation of over 30 reactions, for example fatty acid synthesis and uridine kinase reactions were corrected to maintain proton balance. There are still 59 unbalanced reactions due to multiple allowed charges in BIGG database and complex interrelations. Overall, the mass and charge balance is correct for more than 96% of all reactions as testified by the Memote report (see GitHub repository) and allows for faithful simulations.

The largest connected path we added was connected with the metabolism of erythritol. iUL909 contains four successive reactions that represent the further processing of erythritol. Erythritol is phosphorylated by a kinase to D-erythritol-1-phosphate, which in turn is converted to L-erythrulose-1-phosphate by means of a dehydrogenase and an epimerase. The L-erythrulose-1-phosphate is then split into dihydroxyacetone phosphate and formaldehyde. These cytosolic reactions were necessary to enable the observed growth on the sugar alcohol erythritol. The hydrolysis of many sugars with growth in the phenotype assays is catalyzed in our reconstruction by the maltase enzyme (EC-number 3.2.1.20). Maltase is known to display broad substrate specificity, which was also shown explicitly for *O. polymorpha* [26]. Two reactions were integrated which describe the activities of transketolase and transaldolase in the peroxisome of *O. polymorpha*. These enzymes are essential for the alcoholic fermentation of xylose [27].

### Computational strain engineering tests

**Fig. 3:**
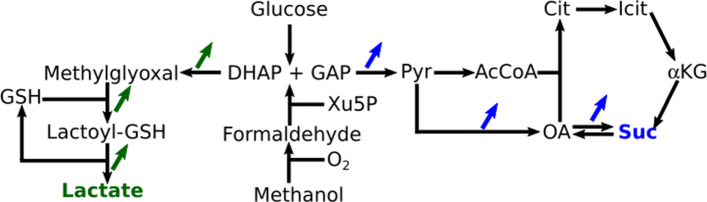
Reaction activation candidates. Reactions to be activated according to FSEOF for overproduction of lactate (green) and succinate (blue). DHAP: Dihydroxyacetonephosphate, GAP: Glyceraldehydephosphate, GSH: Glutathione, Pyr: Pyruvate, AcCoA: Acetyl-CoA, Cit: Citrate, Icit: Isocitrate, *α*KG: *α*Ketoglutarate, Suc: Succinate, OA: Oxaloacetate

Overproduction of important platform chemicals can be achieved with a limited number of genetic manipulations. We chose lactate and succinate as target compounds from methanol and glucose, and applied the FSEOF approach to identify reactions whose increased activity stimulates target production [28]. The optimized synthesis in iUL909 for lactate is routed via methylglyoxal generated from dihydroxyacetone phosphate [29]. Hence, stimulation of the glyoxalase system is predicted to enhance production (Fig. 3, green). Succinate production was increased in silico when the reactions of the lower glycolysis were more active, e.g., glyceraldehyde-3-phosphate dehydrogenase, pyruvate kinase, and pyruvate carboxylase (Fig. 3, blue). The strategies are similar for glucose and methanol. It is interesting to note that low uptake rates of methanol were more sensitive to reactions of the Xyl5P-pathway in the peroxisome. The strategy of reductive TCA cycle stimulation by anaplerotic reactions was experimentally shown to increase succinate production [30].

**Fig. 4:**
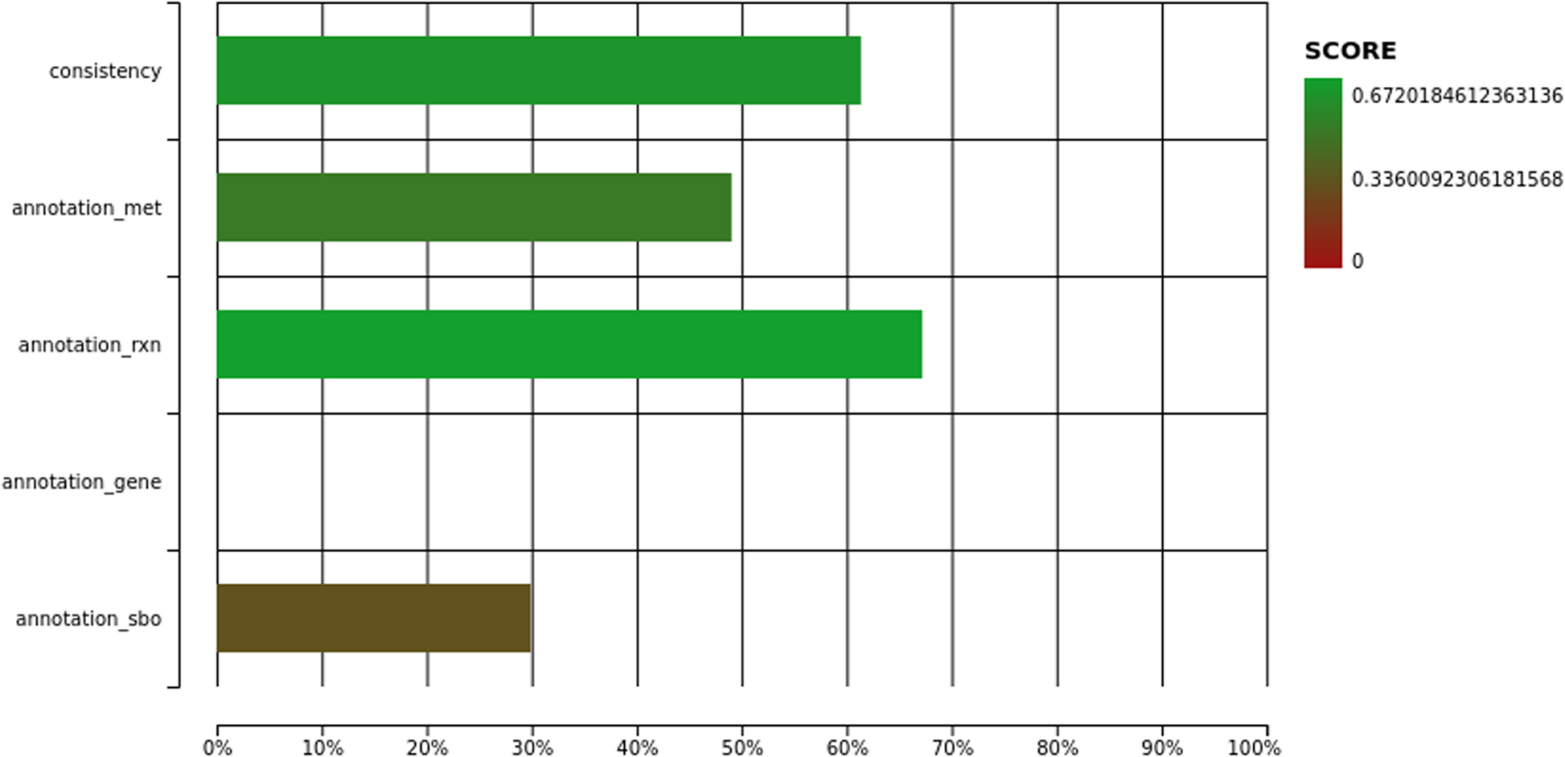
Memote scores of iUL909 regarding quality of reactions, metabolites and annotation degree. The quality of the reconstruction is in total evaluated with 45 % (see also GitHub repository result file). The y-axis represents different model aspects pertaining to structural correctness and the degree of annotation. The consistency is evaluated according to stoichiometric consistency, mass and charge balance, metabolite connectivity and correct exchange reaction definitions. Because approximately 3% of the reactions are not fully balanced, the stoichiometric consistency is reduced and results in a 60% consistency performance. Annotations for metabolites, reactions and SBO-terms were added, however, the gene annotations available in the excel sheet could not integrated to the SBML file. Nonetheless, iUL909 was evaluated nearly double the annotation quality compared to the reference GSMM of iMT1026

iUL909 fulfills key standards for reconstructed models and was tested with Memote and the SBML validator [19, 31]. The SBML validator identifies a valid SBML Level 3v1 file, with flux balance constraints (FBC) in version 2. The detected warnings relate to missing initial concentrations. The total Memote score is 45 % (see Fig. 4, and the GitHub repository for the test result file). This is significantly improved with respect to the reference reconstruction for *K. phaffii* with a score of only 24 %.

## Conclusions

A genome-scale metabolic model of *O. polymorpha* was constructed using homolog identification based on the existing GSMM of *K. phaffii*. The resulting GSMM iUL909 was further tailored for appropriate substrate utilization on the basis of phenotypic microarray and shake flask experiments. Although biomass composition and energetic parameters were adopted from *K. phaffii*, the resulting growth predictions were in good agreement with chemostat experiments for various industrially relevant substrates. The biotechnological applicability was explored by testing overproduction of lactate, succinate, showing that high productivity can be achieved with limited genetic manipulation. Thus, a metabolic model is now available for further strain engineering.

## Methods

### Cultivation experiments

The substrate utilization tests were conducted with the Biolog’s Phenotype Microarray™ plates P1 and P2 with a total of 190 carbon sources. The medium was prepared with the manufacturer’s inoculation medium IFY-0 and the dye mix H according to the guideline. The organism was cultivated in the microarrays in the Growth Profiler (EnzyScreen BV, Heemstede, Netherlands) at 37^∘^C and 150 rpm, growth and respiratory activity was monitored by measuring the optical density (OD) at 490 nm for dye reduction and 750 nm for biomass in a plate reader (Synergy MX, BioTek Instruments Inc., USA) at 0 h and 72 h.

To discriminate growth from non-growth, we considered the distribution of the OD increase for each plate at 490 nm and derived a suitable cut-off. The majority of the OD increases clustered in a normal distribution at the lower OD-end, followed by a long tail with larger OD increases. The tail of the distribution characterizes explicit substrate respiration, while the normal distribution contains metabolized as well as non-utilized substrates. To identify metabolized substrates within the normal distribution, we first separated the normal distribution from the tail with the clear positive substrates. This was achieved by removing all values above the arithmetic mean within a plate. Then, a normal distribution was fitted over the remaining measurements, and one standard deviation above the mean was used as the cut-off for metabolized substrates (see Additional file 2). Moreover, growth was associated with a substrate only when at least 50 % of replicates surpassed the threshold (duplicates for PM1, triplicates for PM2).

Separate-shake flask experiments were performed for the carbon substrates succinate, *α*-ketoglutarate, *α*-ketobutyrate, citrate, fumarate, L-malate and for the carbohydrates D-xylose and D-ribose that failed to grow in the phenotype assays. The cultivation took place in CM2-medium [30] and OD was tested after two, seven and thirteen days (see Additional file 2).

### Computational genome comparison

The *O. polymorpha* genome sequence used for the model construction was based on [7] for strain NCYC 495 retrieved from Uniprot [25]. The *K. phaffii* GSMM iMT1026 [13] was used as reference model for the *O. polymorpha* reconstruction. Replacement of gene-protein-reaction (GPR) relationships was conducted by identification of homologs among *K. phaffii* and *O. polymorpha*. We used the genome sequence of *K. phaffii* GS115, the foundation of iMT1026, extracted from UniProt. The homologs were identified using the software package *ProteinOrtho* using the default parameter settings for homolog detection [32]. ProteinOrtho reports a single best homolog candidate for a *K. phaffii* input protein sequence. *K. phaffii* GPRs were replaced by the *O. polymorpha* homolog, while maintaining isoenzyme and multi-protein complex GPRs, which was possible for 908 GPRs. In the case of failed homolog mappings, manual Blast searches were conducted, and for the reactions of the central carbon metabolism manual comparison of gene annotations were performed with the JGI linked KEGG database of *O. polymorpha* [33] and the MGOB database [23].

The MGOB shows the local genomic organization among methylotrophic yeasts. *K. phaffii* MGOB-IDs of reactions in the central carbon metabolism were derived by querying the MGOB database with KEGG gene IDs. The MGOB-IDs were then used to locate the genes in their genomic context. The alignment with the homologs of all methylotrophic yeasts within MGOB provided information on annotation including subcellular localization. In this way, we could characterize 518 additional genes compared to UniProt. The model completeness and quality was tested with Memote [19]. SBML Level and Version tags were additionally validated with the SBML validator (http://sbml.org/Facilities/Validator).

### Flux balance analysis

Simulations of the GSMM were performed with COBRAv3 on Matlab and with COBRApy. A Jupyter Notebook guide for the simulation of iUL909 and reproduction of experimental data as in Fig. 2 can be downloaded from GitHub (https://github.com/iAMB-RWTH-Aachen/Opol-GSMM). Flux scanning based on enforced objective flux (FSEOF) [28] was performed in Matlab using the functions of COBRA toolbox to identify the targets for overexpression. Briefly, FSEOF was carried out in two stages. In the first stage, the maximum product flux (lactate or succinate) was computed by setting the biomass flux to zero and maximizing the objective function for product formation. In the second stage, the product flux was enforced in steps, and the biomass growth was set as an objective function and maximized. Reactions fluxes increasing monotonically represent targets for over-expression.

## Supplementary Information

**Additional file 1** Supplementary information.

**Additional file 2** Experimental data. Excel sheet with comparison of substrate utilization tests conducted in this paper and Suh et al. [9]. Detailed information on the results of Biolog’s Phenotype Microarray^™^ plates test, namely (i) a list of substrates with abiotic activity, (ii) for plate PM1: OD490/600 and selection of metabolized substrates, (iii) for plate PM1: OD490/600 and selection of metabolized substrates, (iv) shake flask growth results for the eight substrates not grown in phenotype assay (succinate, D-xylose, D-ribose, *α*-ketoglutarate, *α*-ketobutyrate, citrate, fumarate, malate).

**Additional file 3** Genome scale metabolic model details. It contains a list of reactions with only one associated gene-protein-reaction association for which no homolog in *O. polymorpha* was identified. A list of all genes in iMT1026 for which no homologs were identified in *O. polymorpha*. Lists of new reactions and new genes added to the model. A table of literature values for growth rates and substrate uptake rates and finally the results of the FSEOF analysis.

## Data Availability

*O. polymorpha*: NCYC 495 leu1.1 Genome Accession: AECK01000000 The genome scale model iUL909 generated during the current study is available from the corresponding author on reasonable request. A Jupyter Notebook for simulations, the Memote test result, and the reproduction of Fig. 1 is available on GitHub: https://github.com/iAMB-RWTH-Aachen/Opol-GSMM

## Abbreviations

FSEOF: flux scanning based on enforced objective flux
GAM: growth associated maintenance
GPR: gene-protein-reaction relationship
GSMM: genome scale metabolic model
NGAM: non-growth associated maintenance
OD: optical density
SBML: systems biology markup language
TCA: tricarboxylic acid cycle

## Footnotes

1 accessed on 07/31/2018
